# 

**Published:** 1896-02-01

**Authors:** 


					ABSOLUTELY PURE, therefore BEST. February 1st, 1896.
> CADBURYS *w CADBURY'S
- COCOA Tl Jul JPP COCOA
mainable;"?^an?fh6St 81 preSent NO ALKALIES USED.
WW/CH HOSPl.TAJ.
7 NO. 488. Vol. XIX. WITH EXTRA NURSING SUPPLEMENT. ^SSL.SffiS5ESB
Saturday,
Feb. 1st, 1896.
i Oi AUIIIIIII) xvoi uu?. POHL irOQ.
[Registered at the General Post Offioe aa a Newspaper for Monthly Parte, 6d.j post free, 8d
transmission in the United Kingdom and Abroad.] Ditto per Annnm, 8s.6d., post free!

				

